# Navigating the Path to Inclusion: Understanding Barriers and Facilitators to Clinical Trial Participation Among Chinese Older Adults in the United States with Multimorbidity

**DOI:** 10.1007/s11606-024-09162-2

**Published:** 2024-11-04

**Authors:** Derjung M. Tarn, Ruey-Ying Liu, Ting Pun, Janice B. Schwartz

**Affiliations:** 1 Department of Family Medicine, David Geffen School of Medicine at UCLA, University of California, Los Angeles, Los Angeles, CA USA; 2 Department of Sociology, National Chengchi University, Taipei, Taiwan; 3 Patient-Centered Outcomes Research Institute (PCORI) Ambassador, Palo Alto, CA USA; 4 Division of Geriatrics, Department of Medicine and Division of Clinical Pharmacology, Departments of Medicine and Bioengineering and Therapeutic Sciences, University of California, San Francisco, San Francisco, CA USA

**Keywords:** Asian, Chinese, clinical trial participation, aged, focus groups

## Abstract

**Context:**

Older adults with multimorbidity are underrepresented in clinical trials, with enrollment of Asians particularly low.

**Objective:**

Understand perspectives of US Chinese older adults regarding clinical trial participation.

**Study Design and Analysis:**

Focus group interviews analyzed using thematic analysis.

**Setting:**

Community/senior centers, academic health systems in Northern and Southern California, and a nationwide registry of Asian Americans/Pacific Islanders.

**Population Studied:**

Mandarin- and English-speaking Chinese adults aged ≥ 65 years with multimorbidity.

**Outcome Measures:**

Themes related to barriers and facilitators of enrollment in clinical trials of medications.

**Results:**

We conducted 12 focus groups: 7 with non-US-born and 5 with US-born Chinese older adults (*n* = 83 total). Mean age was 74 years (SD = 5.9), 43 (51.8%) were female, and 47 (56.6%) Mandarin-speaking. US-born participants had greater educational attainment than non-US-born participants. Participants took a mean of 6.1 prescriptions (SD = 1.5). Barriers to participation in clinical trials of medications included lack of awareness of/exposure for patients and community-based Chinese physicians, preference for natural/traditional medicine, risk aversion and safety concerns, desire for privacy, and inconvenience. Trusted influences included physicians, hospitals/health systems, Asian/Chinese community centers, and family (for non-US-born participants). Suggestions to enhance participation included using language and culturally concordant materials/personnel, educating community-based Chinese physicians about clinical trials, involving patient-trusted physicians in recruitment, promoting trials on conditions common in Chinese people or for an existing condition, and financial incentives. US-born participants expressed greater understanding and willingness to join trials. All groups attributed low clinical trial enrollment to non-US-born Chinese adults.

**Conclusions:**

Chinese older adults perceived obstacles to clinical trial participation that could be mitigated by involving trusted physicians in recruitment, using language and culturally concordant materials/staff, and educating patients and community-based physicians. Recognition of differences in attitudes among US- and non-US-born Chinese people may be important to tailoring recruitment strategies.

**Supplementary Information:**

The online version contains supplementary material available at 10.1007/s11606-024-09162-2.

## BACKGROUND

Older adults with multiple chronic conditions and those belonging to racial/ethnic minority populations have traditionally been underrepresented in clinical trials.^1–9^ Enrollment of Asian people has been especially low, at only 0–4% of clinical trial participants,^10^ despite Asian people comprising 7.2% of the United States (US) population.^11,12^ Enrollment disparities are concerning because people of different ages and races may differ in factors affecting drug pharmacokinetics, pharmacodynamics, safety, and efficacy.^13–16^

Reported barriers to clinical trial participation in racial/ethnic minority populations include poor awareness, mistrust, logistical complexities, and concerns about adverse events,^17^ with some groups of Asian people reportedly less willing than other racial groups to participate in research.^18^ These conclusions are from studies primarily of non-US-born Asian adults, with analyses of combined age groups or Asian people of multiple origins, and/or included only cancer patients.^19–21^ However, older adults may have distinct concerns due to physical limitations, transportation difficulties, and concerns about medication interactions, and older Asian adults may have unique cultural beliefs preventing participation. Increasingly, researchers have recognized the heterogeneity of racial subgroups and the importance of investigating disparate attitudes that might contribute to health outcomes.^22,23^ Yet few studies on clinical trials have focused on Chinese people, the largest Asian subgroup in the US.^24^ In addition, US-born Asian people comprise over 10% of the older Asian population in the United States^25^ but have largely been absent from studies, though their attitudes may differ from those of non-US-born Asian people.

This study aims to understand perspectives of both US- and non-US-born Chinese older adults and define (1) attitudes/beliefs deterring clinical drug trial participation; (2) facilitators of participation; and (3) recommendations to enhance recruitment.

## METHODS

Focus groups were conducted between November 2022 and April 2023. Participants were 65 years and older, self-identified as “Chinese” or “Taiwanese,” and reported taking 5 or more prescription medications. Participants were recruited from (1) community/senior centers; (2) academic health centers; and (3) California residents of the Collaborative Approach for Asian Americans & Pacific Islanders Research & Education (CARE) registry. At community/senior centers, investigators conducted information sessions and distributed flyers. At the University of California, Los Angeles (UCLA) and the University of California, San Francisco (UCSF), electronic health record data extractions were used to identify potential participants, who were emailed invitations to participate. CARE registry participants were contacted by registrants’ preferred contact method (email or telephone). Recruitment materials were in English and Chinese (traditional and simplified) and explained the study sought perspectives about clinical trial participation. Eligible respondents were purposively sampled based on language (English or Mandarin) and birthplace (US or non-US), with a target of 12 focus groups.

Participants had no prior relationships with investigators. Prior to participation, they were surveyed about their demographics, health conditions, and acculturation.^26^ Ninety-minute in-person (*n* = 6) or Zoom videoconferencing (*n* = 6) focus groups were moderated by a native Mandarin-speaking sociology postdoctoral research fellow (RL) using a semi-structured interview guide (Table 1). A US-born bilingual (English and Mandarin) physician-investigator with focus group expertise (DMT) co-moderated three-quarters of the groups. Participants provided verbal consent and received a $75 gift card. After each focus group, investigators (RL, DMT, and JBS) reflected on major themes and adjusted probes as needed to elicit greater depth of responses.

**Table 1:** Semi-structured Focus Group Interview Questions

Major constructs related to clinical trial participation	Sample question
Knowledge about clinical trials	Have you heard of the term ‘clinical trial’ and have you ever participated in a clinical trial? What comes to mind when you think of a research study (clinical trial) in which you would take a medicine?
Attitudes about clinical trial participation	How do you feel about participating in a research study in which you would take a medicine?
Barriers to participation	In addition to the things that have already come up, what other things might keep you from participating?
Barriers specific to Chinese people	Why do you think so few Chinese people take part in research studies that involve taking a medicine?
Motivators	What are some reasons you might decide to participate in a research study in which you would take a medicine?
Facilitators	What might help increase participation of Chinese people in research studies in which they would take a medicine?

Focus groups were audio recorded, transcribed verbatim, translated into English by a professional transcription company, and verified by investigator RL. Transcripts were analyzed using thematic analysis.^27,28^ Investigators RL, DMT, and JBS independently reviewed transcripts, developed codes to describe discussions, resolved disagreements via consensus, and generated a codebook that briefly defined each code. RL reviewed all transcripts in the language in which focus groups were conducted, while others reviewed English transcripts and English translations of Mandarin transcripts. Subsequently, RL performed focused coding of all transcripts and investigators generated themes, reviewed and adjusted thematic categories together, and compared themes discussed in US- and non-US-born groups. Based on consensus, theoretical saturation of themes^29,30^ was reached. ATLAS.ti 23 (Scientific Software Development, Berlin, Germany) was used for analyses. The UCLA Institutional Review Board (IRB) served as the single-site IRB.

## RESULTS

**Figure 1:**
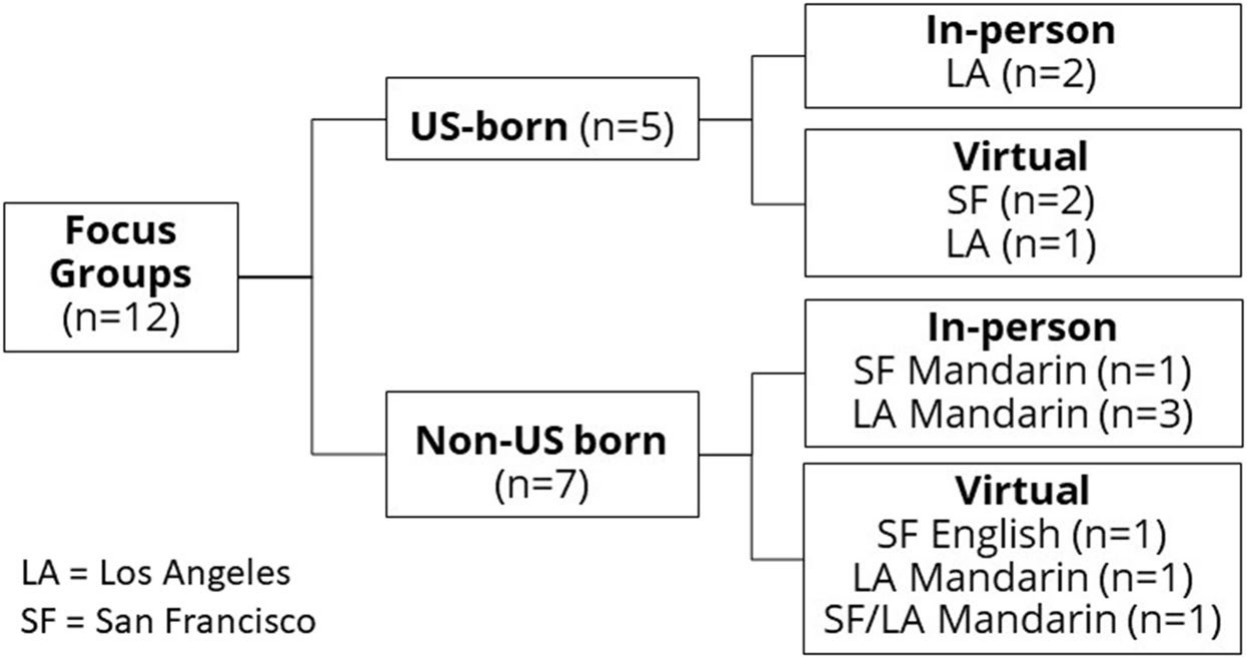
Twelve focus groups comprising 83 participants, seven with non-US-born and five with US-born older Chinese adults. Groups were in Mandarin and English.

We conducted 12 focus groups comprising 83 participants, seven with non-US-born and five with US-born older Chinese adults. Groups were in Mandarin (*n* = 6 with non-US-born participants) and English (*n* = 5 US-born and *n* = 1 non-US-born) (Fig. 1). Participants had a mean age of 74 years (SD = 5.9), took a mean of 6.1 (SD = 1.5) prescription medications, and 43 (51.8%) were female (Table 2). Thirty-seven percent were born in the US, 36% in China, 13% in Taiwan, and the rest in other Asian countries (see Appendix 1 and 2 for additional details).

**Table 2:** Participant Characteristics

Participant characteristics (*n* = 83)	*n* (%) or mean (SD, range)	US-born (*n* = 31)	Non-US-born (*n* = 52)
Age in years, mean (SD, range)	74.0 (5.9, 65–90)	72.6 (4.4, 65–81)	74.9 (6.5, 65–90)
Female, *n* (%)	43 (51.8)	16 (51.6)	27 (51.9)
Birthplace, *n* (%)			
US		31 (37.3)	N/A
Non-US			
China		N/A	30 (36.1)
Taiwan		N/A	11 (13.3)
Other (Vietnam, Indonesia, Myanmar, Korea, Hong Kong, or Macau)		N/A	11 (13.3)
Years in the US, mean (SD, range)		N/A	32.1 (15.8, 4–68)
Participated in Mandarin focus group, *n* (%)		N/A	47 (56.6)
Acculturation score, mean (SD, range)*	1.8 (0.8, 1.0–4.2)	4.4 (0.4, 2.6–4.8)	1.8 (0.8, 1.0–4.2)
Educational level, *n* (%)			
High school diploma or less	20 (24.1)	1 (3.2)	19 (36.5)
Some college or associate degree	15 (18.1)	4 (12.9)	11 (21.2)
Bachelor’s degree	16 (19.3)	8 (25.8)	8 (15.4)
Master’s degree or above	32 (38.5)	18 (58.1)	14 (26.9)
Prior participation in clinical trials, *n* (%)	7 (8.4)	5 (16.1)	2 (3.8)
# prescription medications, mean (SD, range)	6.1 (1.5, 5–12)	6.5 (1.9, 5–12)	5.8 (1.1, 5–9)
# chronic conditions, mean (SD, range)**	4.1 (1.6, 0–9)	3.6 (1.6, 0–7)	4.4 (1.2, 3–9)
Most common chronic conditions, *n* (%)* ±			
Hyperlipidemia	62 (75.6)	24 (77.4)	38 (73.1)
Hypertension	61 (74.4)	21 (67.7)	40 (76.9)
Osteoporosis	35 (42.7)	7 (22.6)	28 (53.8)

^*^Acculturation ranges from 1 to 5, with higher numbers indicating greater acculturation^26^

^**^Self-reported from a list of 16 chronic conditions

± *n* = 82

US-born participants were younger than non-US-born participants (mean age 72.6 (SD = 4.4) versus 74.9 years (SD = 6.5)), attained a higher level of education (83.9% versus 42.3%, respectively, had at least a Bachelor's degree), and had greater clinical trial participation (16.1% versus 3.8%). US-born participants reported fewer medical conditions than non-US-born participants (mean 3.6 (SD = 1.6) versus 4.4 (SD = 1.2)) but reported more prescription medications (mean 6.5 (SD = 1.9) versus 5.8 (SD = 1.1)). Almost all participants had previously heard the term “clinical trial.”

Comparisons of the US- and non-US-born focus groups demonstrated that US-born participants expressed greater knowledge and willingness to join clinical trials. The groups otherwise mostly touched on similar themes, with some differences noted below. All groups attributed low clinical trial enrollment to non-US-born Chinese people with more limited health literacy and primarily referenced barriers and facilitators relating to that group. Major themes related to attitudes and beliefs around clinical trial participation are described below and in Table 3. As applicable, Table 3 also provides examples of subthemes.

**Table 3:** Major Themes and Example Quotes

Major themes and subthemes	Example quotes
Lack of awareness/exposure to clinical trials	
Patients lack awareness	“You do not know about us and we do not know about you. In this case, it is certain that the Chinese are less involved.” (Female2/FG3/translated)
Patients have never been asked	“In this room, 4 out of 4 people have never been asked. I think 4 out of 4 people would consider participating in a clinical trial.” (Male1/FG9/US-born)
Community-based physicians lack awareness	“…most physicians in private practice aren’t necessarily aware of a lot of things that are going on in the teaching facilities and new drugs…” (Male2/FG9/US-born)
Heterogeneous views on clinical trial participation	
Generational differences	“…maybe the trend has been that more especially Chinese Americans will participate in clinical trials because I think just from the feedback here, the influence of the culture that we grew up in in the United States…made us less shy.” (Female3/FG12/US-born)
Differences based on country of origin	“… the new Chinese are probably more modern than some of the Chinese in America. Compared to my dad’s age generation and my age generation, even those who are coming from Taiwan and from Hong Kong…I think in the past maybe they were more traditional.” (Male2/FG10/US-born)
Preference for natural/traditional medicine	
	“Asian elders are used to tea or whatever or acupuncture or different things, they have a distrust of anything foreign going into their body that they can’t pronounce, that they don’t understand.” (Female1/FG9/US-born)
Risk aversion and safety concerns	
Risk aversion	“…most of us may take a reserved approach, especially the elderly…the Westerners may be more open-minded.” (Female2/FG5/translated)
Fear of adverse outcomes	“Sometimes, the harm [from trials] is minor, and they can make it up to you, but life is not something that can be made up.” (Male1/FG1/translated)
Drug interactions	“The people you have invited to join this focus group are all taking many kinds of medicines, and that’s why we are reluctant to participate in trials.” (Male2/FG6/translated)
Concerns about receiving placebo medication	“…one reluctance of some folks for clinical trials is that you have this disease and you’re getting the placebo…Why would I do it, right, based on my disease might progress and get worse?” (Male4/FG10/US-born)
Desire for privacy	
	“A lot of people have this mentality, ‘I don’t want people to know’ [about my medical problems].” (Female5/FG8/US-born)
Inconvenience/physical limitations can preclude participation	
Mobility	“My hands are numb and swollen, as well as my eyes, and I can hardly walk.” (Female2/FG2/translated)
Urinary incontinence	“I’ll leave [this discussion] for 2 min, okay?…I’ll go to the bathroom, okay?” (Female2/FG6/translated)
Time constraints	“I work and I hate not having the availability to do the things I need to do for work to get done. It would be an interference with my daily routine.” (Male3/FG9/US-born)
Transportation: Do not drive	“You have to ask your kids to drive you there, and they have to ask for leave. It would be indeed very troublesome.” (Female4/FG2/translated)
Transportation: Distance/travel time	“… usually the site is too far, and I will have to drive over half an hour. That’s not quite convenient for me.” (Female2/FG12/US-born)
Like to avoid hassle	“Asians…try to avoid all things that are a hassle…they see it as a hassle…It’s just something extra they don’t want to do.” (Female1/FG8/US-born)
Motivators include getting treatment as a last resort or for perceived personal or cultural relevance	
As last resort	“A lot of times, especially at our age when you’re looking at clinical trials, it’s because you’ve run out of options…you have no other answers.” (Male 2/FG11/US-born)
For a personal medical condition	“I’ve been a diabetes patient for close to 35 years, so anything they can improve the diabetes situation, I will be more than happy to participate.” (Male3/FG7/non-US-born/English)
For a condition common in Chinese people	“What comes to mind in Asian diseases is hypertension. I may probably try to participate to find out why Asians tend to have hypertension and maybe some medication can help reduce it…” (Male2/FG10/US-born)
Incentives are important	
To cover transportation	“…usually if you pay more it’s an incentive and people are more willing, [laughs] hate to say that but that’s a fact.” (Female5/FG12/US-born)
Defray medication costs	(On participating in a clinical trial): “First and foremost, it was that they were going to cover the cost of the drugs. The oncologist did say if I weren’t part of the clinical trials, it was going to be terribly expensive. I got no monetary compensation for the trial itself. I was going to have to go through chemotherapy anyway, so it made sense to try this.” (Male 2/FG8/US-born)
Major recommendations for promoting clinical trial participation	
Hearing from other Chinese people	“It might be good that the information come from other Chinese people because I think there might be a tendency to give more store to somebody who’s Chinese.” (Female5/FG8/US-born)
Using both language-concordant and culturally sensitive recruitment materials	“…it needs to be culturally sensitive in their language and their culture. Without that, you will never succeed regardless of their faith in traditional medicine or Western medicine.” (Male2/FG7/non-US-born/English)
Tailor strategies based on Chinese subgroup	“…if you’re going after, let’s say, Chinese Americans, which location, which population are you going after, and then how do you reach out to them.” (Male6/FG10/US-born)

### Lack of Awareness/Exposure to Clinical Trials

Participants mostly attributed underrepresentation of Chinese people in clinical trials to poor awareness of opportunities for participation. One participant observed: “…not a lot of the Asian community is really aware of [clinical trials]…I’ll be 76, we didn’t really have an awareness that clinical trials exist…it’s a communication gap” (Female1/FG9/US-born). As another participant remarked, Chinese people do not oppose participation: “It’s that we are not given access to, not that we refuse to join” (Male1/FG5/translated from Mandarin). Similarly, another noted: “I’ve never been invited for a clinical trial. I don’t know anyone in my group has been invited” (Male7/FG1/translated).

Participants in several groups remarked that community-based physicians, particularly those in primarily Chinese communities, may lack knowledge about clinical trials, as exemplified in one exchange:

Male4: If you go to your primary doctor in Chinatown, they probably don’t even know about clinical trials.

Male7: That’s right. If your physician is not part of a university hospital…you will never be offered clinical trials. (FG10/US-born)

### Heterogeneous Views on Clinical Trial Participation Based on Generation, Country of Origin

Several focus groups discussed differences among Chinese subgroups. One proclaimed that: “We want to be seen as individuals as opposed to a giant group of ‘all Chinese people would think to do this or feel this way’” (Female5/FG8/US-born). Another participant supported this perspective:

I think it is a generational thing–you’d have to target each Chinese American that you’re talking about before you just lump them into one… you can’t just do a very stereotypical, “Chinese Americans don’t do this because--” I think that would not be appropriate. (Female4/FG12/US-born)

A common sentiment was that US-born Chinese people (and those who immigrated early in life) are more open to clinical trial participation. Commenting about younger generations, a first-generation participant observed:

They are more willing to [participate]. We can see that the young generation born here thinks differently from us, the older immigrants. (Female1/FG4/translated)

Another speculated that both generation and country of origin are influential:

I think there’s various different subgroups among the older Chinese, and I’m guessing that American-born Chinese, they’d probably have a higher participation rate than immigrant Chinese…There’s also a difference between the various subgroups [of Chinese people]. Like the mainland Chinese used to come recently. Actually some of them are well-off and their level of knowledge and trust is the same as an American-born. (Male2/FG8/US-born)

### Preference for Natural/Traditional Medicine

A common theme was a preference for natural (traditional) treatments. Participants voiced that first-generation Chinese people may mistrust Western medicine, and consequently decline participation in a trial of medications. As one participant articulated:

Chinese people believe that our traditional Chinese medicine is more reliable, whereas Western medicines are all made of chemicals…for a long time, we have been so used to believing that Chinese medicines are safer while Western medicines are made of chemicals, like those made in laboratories, so that makes us more frightened…We all have this kind of belief. (Female1/FG4/translated)

Others echoed these thoughts, for example: “…my Mom didn’t believe in western medicines that much because she said that they’re too strong, they make you sick” (Female6/FG12/US-born).

### Risk Aversion and Safety Concerns

Participants in all groups feared being a “guinea pig” or a “lab rat.” Some Mandarin-speaking participants commented about lacking “courage” to face potential clinical trial risks. As discussed in one focus group:

Female2: [Chinese people] tend to be more cautious and don’t want to take risks…

Male4: Because life is so precious to them. They traveled tens of thousands of miles to the United States.

Female1: Chinese people have suffered too much.

Female2: They are more cautious.

Male4: So they cherish the chance of living here, and being more cautious. This is where the Chinese are different from [Westerners]. (FG1/translated)

Risk aversion was discussed in several focus groups, and as one participant commented: “…a lot of Chinese people–my parents are from China, and they’re very averse to risk…they don’t want to take that risk” (Male1/FG10/US-born).

All groups had concerns about trial medication, adverse outcomes, and drug interactions. As one participant conveyed: “…I will not participate in clinical trial if the new drug interferes with the efficacy of the drugs I’m taking now” (Male1/FG6/translated). Some US-born participants worried about the consequences of being randomized to the placebo arm of a clinical trial.

### Desire for Privacy

Privacy was mentioned by US-born participants, who asserted that they were raised to keep to themselves, for example:

My parents were immigrants, Chinese immigrants…I know that growing up, they really instilled on us…don’t get involved, and don’t give out any information you don’t need to. (Female2/FG8/US-born)

Participants hypothesized that this desire for privacy may deter Chinese people from disclosing their health conditions and participating in clinical trials. As one participant shared:

Your own body is your own body and it’s sacred, so you don’t want to share with the world what’s going on, and you’re taught to be a little bit more private. You just don’t have these open discussions as to your ailments, especially if it’s not comfortable to talk about…You don’t share with your best friend, so why would you share with a stranger? (Female4/FG11/US-born)

### Inconvenience/Physical Limitations Can Preclude Participation

Other barriers to participation included physical limitations such as mobility and urinary incontinence. Time constraints were mentioned by those who were still working, and distance/travel time was a consideration for some. Inconvenience was a concern, for example:

…you also have to think about if the trial is going to affect your life, or any possible restrictions on your diet or on your activities during the trial…It would be inconvenient if there are a lot of changes. (Female1/FG2/translated)

### Motivators Included Getting Treatment as a Last Resort or for Perceived Personal or Cultural Relevance

Participants in all groups remarked that they would consider clinical trial participation if they exhausted other treatment options, or as multiple Mandarin-speaking participants mentioned, “treating a dead horse as if it were alive,” an expression denoting making every possible effort in a hopeless situation. Others would consider participating for a personal condition, for example: “I’ve been a diabetes patient…anything they can improve the diabetes situation, I will be more than happy to participate” (Male3/FG7/non-US-born/English). Some were willing to participate if a trial studied diseases common in Asians, for example:

…for a serious disease like malaria that has an impact on Asian populations…for several other infectious diseases that affect people in Asia…I might be more willing to volunteer. (Male5/FG10/US-born)

One participant speculated: “Maybe stressing that it would help other Chinese as well, that–not very many Chinese enter clinical studies and how important it is for other Chinese” (Female3/FG11/US-born) would increase participation.

### Incentives Are Important

When asked directly, participants conveyed that incentives were mostly unimportant. Yet they felt parking and gas should be covered, and some assumed transportation would be provided. One group joked that the greater the incentives, the greater the participation. A minority discussed receiving a trial medication at free or reduced cost: “Let’s say, there’s a new drug for cancer, but it costs $100 a day. If a new trial can bring the cost down, I would be willing to give it a try” (Male2/FG6/translated).

### Major Recommendations for Promoting Clinical Trial Participation

Both US- and non-US-born participants wanted to hear about clinical trials from other Asians. One US-born participant remarked:

It gave me more confidence that there was an Asian involved [in this study] versus a person of another ethnicity. It makes it more comfortable. I’ve always felt perhaps more comfortable among Asians if they’re going to be getting very personal. Certainly, I looked at the names of the people involved in [this] study and I was more amenable to participating when I saw Asian names. (Male1/FG11/US-born)

Others lamented the lack of information in Chinese and discussed the need for both language-concordant and culturally sensitive materials, because direct translation may result in unintended meanings. Others voiced the importance of forming: “a bridge between the Western medicine and Chinese medicine and a bridge between the generations” (Female3/FG12/US-born).

Multiple participants stated that their physician’s recommendation could prompt participation. Other trusted voices included hospitals/academic health centers and Asian/Chinese healthcare organizations. Participants felt that community/senior centers were a good venue for obtaining information, and often mentioned group presentations, newspaper, television, and advertising (Appendix Table 3).

Non-US-born groups with less English proficiency valued support from their adult children, while few US-born participants mentioned their adult children. US-born groups brainstormed more strategies for outreach than non-US-born groups, though their suggestions primarily focused on recruitment of non-US-born older adults.

## DISCUSSION

Focus groups with Chinese older adults with multimorbidity revealed specific barriers to clinical trial participation that include cultural influences on attitudes and poor knowledge of opportunities to participate and that potential differences in willingness to participate in clinical trials may reflect generation and country of birth. US-born participants expressed greater willingness to participate in clinical trials, observed greater hesitancy in first-generation Chinese immigrants, and attributed underrepresentation of Chinese people in clinical trials to the lack of participation by non-US-born Chinese. Our findings demonstrate that unlike younger Asians reported to be less willing to participate in research studies than other races/ethnicities,^18^ subgroups of older adults may participate if invited. While a preference for traditional or natural medicine was stated, almost all participants, including those stating skepticism about Western medications, reported taking at least 5 prescription medications, suggesting willingness to use medications for existing health conditions. Participants were also willing to consider clinical trials when other treatment options had been exhausted, for personal health conditions, or for trials addressing conditions common in Asians.

Major barriers to clinical drug trial participation included paucity of invitations, risk aversion and safety concerns, and a desire for privacy regarding medical problems. In contrast to results of a prior national survey showing that only 20% of Asians had ever heard of a clinical trial,^31^ the majority of our participants had heard the term clinical trial but many (particularly non-US-born participants) were unaware of specific details regarding trial design or procedures or of clinical trial opportunities, and most had never participated.

Participants strongly valued physician recommendations, matching results of national surveys of US adults of all ages and of older adults, in which over 75% preferred hearing about clinical trials from their physician or other healthcare provider.^32,33^ Yet as observed by our study participants, community-based physicians (particularly those in predominantly Chinese communities) may lack information about clinical trials. Indeed, research reveals that primary care physicians, regardless of practice location or racial and ethnic background, may be unaware of clinical trial opportunities, and have significant time constraints during office visits^34^ that may prevent discussing trials. Investigators could engage more with community-based physicians about ongoing trials, conduct educational sessions for physicians with continuing medication education credit, and engage community-based physicians in recruitment efforts. Our participants also gave importance to recommendations from hospitals/health systems and community centers, implying that efforts to engage these entities may promote clinical trial participation.

Suggestions to enhance Chinese older adult participation in clinical trials mirror recommendations of recent National Academies of Science, Engineering, and Medicine reports.^35,36^ Specifically, to have investigators and research staff that are of the same race/ethnicity, communications in patients’ preferred language, engagement of the community, and education about the importance of clinical trials.^35,36^ Outreach using previous clinical trial participants of the same race/ethnicity to share their experiences may allay patient concerns about participation. Future studies could investigate whether clinical trials that employed any of the strategies suggested in the focus groups achieved greater success in recruiting Chinese participants.

Study limitations include those associated with focus group methodology which elucidates a range of themes, rather than the frequency with which participants hold the perspectives raised in the groups. Participants were from California and may not reflect perspectives of Chinese older adults in other parts of the country or Chinese older adults who speak only Cantonese. Questions asking US-born focus group participants to reflect on “Chinese” people may have led them to focus their thoughts on non-US-born Chinese.

To the best of our knowledge, this is the only study to conduct separate focus group interviews with US- and non-US-born Chinese participants in their chosen language, thus providing an understanding of the perspectives of different generations of Chinese older adults. Additional studies are needed to investigate whether and which perspectives are generalizable to all Asian populations, other immigrant populations, or all older adults. The findings suggest that combining Asian and even Chinese subgroups into a single group may be inappropriate, and that strategies may need to change as US-born Chinese adults age. Chinese older adults come from different generations and countries of origin and have different language capabilities, making it important for investigators to engage with Chinese community members who can provide insight into the heterogeneous makeup and dynamics of the Chinese population. Interventions have decreased disparities in participation among Black or African American and Hispanic or Latino people,^5^ but work is still needed for Asian people. Effective strategies to enhance clinical trial participation in older US Chinese adults should include tailoring of recruitment strategies to the needs of Chinese people and will be most effective if they match people to trials targeting conditions and diseases that are prevalent in this population and engage with community physicians and organizations.

## Supplementary Information

Below is the link to the electronic supplementary material.

Supplementary file1 (DOCX 37.4 KB)

## Data Availability

With IRB approval, deidentified transcripts of focus groups are available upon request for research purposes.

## Declarations:

### Disclaimer:

This article reflects the views of the authors and should not be construed to represent the FDA or NIH’s views or policies.
