# Supplementary material for: Navigating the Path to Inclusion: Understanding Barriers and Facilitators to Clinical Trial Participation Among Chinese Older Adults in the United States with Multimorbidity
Source: J Gen Intern Med. 2024 Nov 4;40(2):393–401. doi: 10.1007/s11606-024-09162-2 (PMC11802972; doi:10.1007/s11606-024-09162-2)
Supplement: Supplementary file 1 — Supplementary file1 (DOCX 37.4 KB) [file 11606_2024_9162_MOESM1_ESM.docx]

**Appendix Table 1. Non-US-Born Focus Group Participant Characteristics**

| **Participant Characteristic** | **FG1** | **FG2** | **FG3** | **FG4** | **FG5** | **FG6** | **FG7** |
| --- | --- | --- | --- | --- | --- | --- | --- |
| n | 9 | 10 | 8 | 6 | 7 | 7 | 5 |
| FG language | Mandarin | Mandarin | Mandarin | Mandarin | Mandarin | Mandarin | English |
| FG location * | Los Angeles | Los Angeles | Los Angeles | San Francisco | SF+LA | Los Angeles | San Francisco |
| FG mode | In Person | In Person | In Person | In Person | Virtual | Virtual | Virtual |
| Age in years, mean (SD, range) | 75.8 (7.0, 66-90) | 74.5 (6.6, 65-83) | 77.9 (5.1, 68-83) | 73.7 (4.5, 66-79) | 72.3 (8.6, 65-84) | 73.9 (6.0, 65-79) | 75.8 (8.2, 65-82) |
| Female, n (%) | 3 (33.3) | 7 (70.0) | 5(62.5) | 2 (33.3) | 5 (71.4) | 3 (42.9) | 2 (40.0) |
| Birthplace, n (%) |  |  |  |  |  |  |  |
| China | 5 (55.6) | 5 (50.0) | 5 (62.5) | 3 (50.0) | 5 (71.4) | 5 (71.4) | 2 (40.0) |
| Taiwan | 3 (33.3) | 2 (20.0) | 2 (25.0) | 0 (0) | 2 (28.6) | 2 (28.6) | 0 (0) |
| Other (Vietnam, Indonesia, Myanmar, Korea, Hong Kong, or Macau) | 1 (11.1) | 3 (30.0) | 1 (12.5) | 3 (50.0) | 0 (0) | 0 (0) | 3 (60.0) |
| Educational level, n (%) |  |  |  |  |  |  |  |
| High school diploma or less | 3 (33.3) | 8 (80.0) | 5 (62.5) | 3 (50.0) | 0 (0) | 0 (0) | 0 (0) |
| Some college or associate degree | 3 (33.3) | 1 (10.0) | 1 (12.5) | 1 (16.7) | 3 (42.9) | 1 (14.3) | 1 (20.0) |
| Bachelor’s degree | 1 (11.1) | 1 (10.0) | 2 (25.0) | 2 (33.3) | 1 (14.3) | 0 (0) | 1 (20.0) |
| Master’s degree or above | 2 (22.2) | 0 (0) | 0 (0) | 0 (0) | 3 (42.9) | 6 (85.7) | 3 (60.0) |
| Prior participation in clinical trials, n (%) | 0 (0) | 0 (0) | 1 (12.5) | 0 (0) | 0 (0) | 1 (14.3) | 1 (20.0) |
| # prescription medications, mean (SD, range) | 5.1 (0.3, 5-6) | 5.5 (0.8, 5-7) | 5.5 (0.8, 5-7) | 6.7 (1.4, 5-8) | 6.3 (1.1, 5-8) | 5.6 (1.5, 5-9) | 7.0 (1.2, 6-9) |
| # chronic conditions, mean (SD, range) ** | 4.3 (1.7, 2-6) | 3.9 (0.9, 3-5) | 4.1 (1.6, 2-6) | 4.8 (1.9, 3-8) | 4.3 (0.8, 3-5) | 4.0 (0.8, 3-5) | 6.0 (2.6, 3-9) |
| Years in the US, mean (SD, range) | 26.9 (11.3, 11-42) | 25.4 (16.7, 6-56) | 29.0 (13.3, 12-41) | 29.2 (16.3, 4-45) | 32.5 (14.5, 13-53) | 39.7 (14.8, 12-53) | 51.8 (14.6, 29-68) |
| Acculturation score, mean (SD, range) ± | 1.4 (0.3, 1.1-1.9) | 1.4 (0.4, 1.0-2.4) | 1.3 (0.2, 1.2-1.7) | 2.0 (1.0, 1.2-3.5) | 2.3 (1.0, 1.3-4.2) | 2.3 (0.9, 1.5-3.9) | 3.2 (0.4, 2.9-3.8) |
| * For virtual focus groups, location indicates where participants were recruited  ** Self-reported from a list of 16 chronic conditions  ± Acculturation ranges from 1-5, with higher numbers indicating greater acculturation | | | | | | | |

**Appendix Table 2. US-Born Focus Group Participant Characteristics**

| **Participant Characteristic** | **FG8** | **FG9** | **FG10** | **FG11** | **FG12** |
| --- | --- | --- | --- | --- | --- |
| n | 7 | 4 | 7 | 6 | 7 |
| FG language | English | English | English | English | English |
| FG location * | Los Angeles | Los Angeles | San Francisco | San Francisco | SF+LA |
| FG mode | In Person | In Person | Virtual | Virtual | Virtual |
| Age in years, mean (SD, range) | 71.0 (3.7, 67-75) | 71.5 (5.5, 65-77) | 73.0 (4.8, 67-81) | 72.0 (3.0, 69-77) | 75.0 (5.0, 68-80) |
| Female, n (%) | 5 (71.4) | 1 (25.0) | 0 (0) | 4 (66.7) | 6 (85.7) |
| Educational level, n (%) |  |  |  |  |  |
| High school diploma or less | 0 (0) | 1 (25.0) | 0 (0) | 0 (0) | 0 (0) |
| Some college/associate degree | 0 (0) | 0 (0) | 2 (28.6) | 0 (0) | 2 (28.6) |
| Bachelor’s degree | 4 (57.1) | 1 (25.0) | 0 (0) | 2 (33.3) | 1 (14.3) |
| Master’s degree or above | 3 (42.9) | 2 (50.0) | 5 (71.4) | 4 (66.7) | 4 (57.1) |
| Prior participation in clinical trials, n (%) | 1 (14.3) | 0 (0) | 1 (14.2) | 1 (16.7) | 2 (28.6) |
| # prescription medications, mean (SD, range) | 5.6 (1.5, 5-9) | 5.5 (1.4, 5-8) | 7.1 (2.0, 5-10) | 7.0 (2.6, 5-12) | 6.9 (1.8, 5-10) |
| # chronic conditions, mean (SD, range) ** | 4.0 (1.5, 3-7) | 3.3 (0.5, 3-4) | 3.0 (2.2, 0-7) | 4.2 (1.0, 3-6) | 3.7 (1.8, 0-5) |
| Acculturation score, mean (SD, range) ± | 4.5 (0.3, 4.1-4.8) | 4.7 (0.1, 4.6-4.8) | 4.5 (0.3, 4.1-4.8) | 4.5 (0.2, 4.1-4.7) | 3.9 (0.7, 2.6-4.5) |
| * For virtual focus groups, location indicates where participants were recruited  ** Self-reported from a list of 16 chronic conditions  ± Acculturation ranges from 1-5, with higher numbers indicating greater acculturation | | | | | |

**Appendix Table 3. Number and % of Focus Groups in which Recruitment Medium/ Source/Location was Suggested, by Non-US- Versus US-Born Groups***

| **Medium** | **Number (%) of Non-US-Born Focus Groups (total n=7);** | **Number (%) of US-Born Focus Groups (total n=5)** |
| --- | --- | --- |
| Group presentations | 5 (71) | 3 (60) |
| Mass media (general) | 4 (57) | 3 (60) |
| Chinese language newspaper (e.g., World Journal) | 2 (29) | 2 (40) |
| Chinese television | 2 (29) | 2 (40) |
| Chinese radio | 1 (14) | 1 (20) |
| Mainstream media (e.g., NPR, 60 Minutes, morning talk shows) | 1 (14) | 1 (20) |
| Printed material (flyers, brochures, etc.) | 2 (29) | 2 (40) |
| Word of mouth | 2 (29) | 2 (40) |
| Video conferencing | 2 (29) | 1 (20) |
| One-on-one presentations | 1 (14) |  |
| Chinese New Year parade float |  | 1 (20) |
| **Source** |  |  |
| Physician (primary care, specialist) | 7 (100) | 5 (100) |
| Physicians caring for the Chinese community | 2 (29) | 4 (80) |
| Hospital / health system |  |  |
| Teaching hospital (UCSF, UCLA) | 4 (57) | 4 (80) |
| Asian/Chinese healthcare organization |  | 3 (60) |
| Chinese hospital | 2 (29) | 2 (40) |
| Asian/Chinese organization (non-healthcare) | 1 (14) | 1 (20) |
| Family / friends | 3 (43) | 1 (20) |
| Prior study participant (Chinese) | 2 (29) | 1 (20) |
| Pharmacist | 1 (14) | 1 (20) |
| Celebrity (e.g., Academy Award winners such as Michelle Yeoh, Keanu Reeves, congressional representatives) |  | 1 (20) |
| Bus tour group leaders (e.g, to Las Vegas, Reno) |  | 1 (20) |
| Asian medical, dental, pharmacy, and undergraduate students |  | 1 (20) |
| **Location of talks or information** |  |  |
| Community / senior center | 3 (43) | 3 (60) |
| Website (perhaps with live chat, blog) | 2 (29) | 1 (20) |
| Email | 1 (14) | 2 (40) |
| Social media (e.g., Facebook, WhatsApp, WeChat) | 1 (14) | 2 (40) |
| Health fair / street fair |  | 2 (40) |
| YouTube | 1 (14) |  |
| Supermarket |  | 1 (20) |
